# Subjective Well-being, Status Identity, and Intergenerational Relations among the Elderly

**DOI:** 10.1093/geroni/igab046.2911

**Published:** 2021-12-17

**Authors:** Jieming Chen

**Affiliations:** Texas A&M University - Kingsville, KIngsville, Texas, United States

## Abstract

This study investigates the influences of intergenerational relations on the subjective wellbeing and status identity of the elderly population in China. The project draws insights from the studies of social mobility and stratification, and that of family relations and old age support. Because of widespread exchange of economic resources across generations and strong sense of connectedness among parent and adult children families that continue to exist in Chinese society today, we hypothesize that older parents’ subjective sense of well-being and evaluation of their socioeconomic statuses are positively related with the socioeconomic conditions of their grown children, and the strength of the such relations with them. The study used the data from the 2013 China General Social Survey (CGSS), and the results provide fairly strong support to the hypotheses. The implications of the results on age-based stratification are discussed.

